# The association between hypoalbuminemia and risk of death due to cancer and vascular disease in individuals aged 65 years and older: findings from the prospective Moli-sani cohort study

**DOI:** 10.1016/j.eclinm.2024.102627

**Published:** 2024-05-08

**Authors:** Augusto Di Castelnuovo, Marialaura Bonaccio, Simona Costanzo, Amalia De Curtis, Sara Magnacca, Mariarosaria Persichillo, Teresa Panzera, Francesca Bracone, Pasquale Pignatelli, Roberto Carnevale, Chiara Cerletti, Maria Benedetta Donati, Giovanni de Gaetano, Licia Iacoviello, Francesco Violi, Moli-sani Study Investigators, Licia Iacoviello, Giovanni de Gaetano, Maria Benedetta Donati, Chiara Cerletti, Marialaura Bonaccio, Americo Bonanni, Simona Costanzo, Amalia De Curtis, Augusto Di Castelnuovo, Alessandro Gialluisi, Francesco Gianfagna, Mariarosaria Persichillo, Teresa Di Prospero, Jos Vermylen, Renzo Pegoraro, Antonio Spagnolo, Deodato Assanelli, Livia Rago, Simona Costanzo, Marco Olivieri, Sabatino Orlandi, Teresa Panzera, Augusto Di Castelnuovo, Marialaura Bonaccio, Simona Costanzo, Simona Esposito, Alessandro Gialluisi, Anwal Ghulam, Francesco Gianfagna, Roberta Parisi, Antonietta Pepe, Emilia Ruggiero, Francesca Bracone, Sukshma Sharma, Amalia De Curtis, Concetta Civitillo, Alisia Cretella, Sara Magnacca, Fabrizia Noro, Mariarosaria Persichillo, Francesca Bracone, Giuseppe Di Costanzo, Sabrina Franciosa, Martina Morelli, Teresa Panzera, Americo Bonanni

**Affiliations:** a Department of Epidemiology and Prevention, IRCCS Neuromed, Pozzilli, Italy; b Department of Clinical Internal, Anaesthesiologic and Cardiovascular Sciences, Sapienza University of Rome, Rome, Italy; c Department of Medical-Surgical Sciences and Biotechnologies, Sapienza University of Rome, Rome, Italy; d IRCCS Neuromed, Pozzilli, Italy; e Department of Medicine and Surgery, LUM University, Casamassima, Italy

**Keywords:** Albumin, Mortality, Cancer, Vascular disease

## Abstract

**Background:**

Serum albumin is inversely associated with overall mortality, but its association with specific causes of death remains uncertain. This study aims to investigate whether hypoalbuminemia, defined as serum albumin levels ≤35 g/L, is associated with mortality specifically attributed to cancer and/or vascular diseases.

**Methods:**

Serum albumin levels were measured in the population-based, prospective cohort of the Moli-sani study, established between 2005 and 2010. Hypoalbuminemia was defined as serum albumin levels ≤35 g/L. Cause-specific mortality was assessed using the validated Italian mortality registry and coded according to the International Classification of Diseases, Revision 9. Over a median follow-up period of 13.1 years, the relationship between serum albumin and mortality, adjusted for covariates, was investigated using competing-risk survival analysis.

**Findings:**

The analysed cohort comprised 17,930 individuals aged ≥35 years, of whom 8445 were men (47.1%). The mean age was 54 years (standard deviation (SD) = 11 years), with 3299 individuals (18.4%) aged older than 65 years. All participants had C-reactive protein levels <10 mg/L and no history of liver, renal, cardiovascular, or cancer disease. Hypoalbuminemia was found in 406 individuals (2.3%). The study documented a total of 1428 deaths, with 574 attributed to cancer and 464 to vascular causes. Hypoalbuminemia was independently associated with mortality when compared to serum albumin >40 g/L (Hazard Ratio (HR) = 1.61, 95% Confidence Interval: 1.21–2.13). A decrease of 1-SD in serum albumin levels corresponded to HR of 1.16 (1.09–1.22), 1.16 (1.05–1.28), and 1.13 (1.03–1.23) for total, vascular and cancer mortality, respectively. Upon stratifying by age, hypoalbuminemia was associated with total mortality solely in those aged ≥65 years (HR = 1.83; 1.33–2.50) but not in the <65 years group (HR = 1.03; 0.53–2.00; P < 0.0001 for difference). Similar age-related patterns emerged for vascular death (per 1-SD decrease HR = 1.19; 1.07–1.33 in individuals ≥65 years and HR = 1.05; 0.86–1.29 in individuals <65 years) and cancer mortality (HR = 1.15; 1.02–1.30; ≥65 years and HR = 1.08; 0.96–1.23; <65 years).

**Interpretation:**

Individuals ≥65 years old with serum albumin levels ≤35 g/L are at higher risk of total, cancer, and vascular mortality.

**Funding:**

This paper was developed within the project funded by Next Generation EU–“Age-It - Ageing well in an ageing society” project (PE0000015), National Recovery and Resilience Plan (NRRP)–PE8–Mission 4, C2, Intervention 1.3.

**Research in context**

**Evidence before this study**

Previous studies indicated an inverse correlation between serum albumin and mortality but often failed to account for confounding factors such as renal and liver diseases, inflammation, and malnutrition. However, there is a lack of comprehensive analysis excluding these confounders, necessitating further investigation to establish a robust association and define a universal serum albumin cut-off for identifying high-risk individuals.

**Added value of this study**

Our study contributes novel insights into the relationship between hypoalbuminemia and mortality in a general adult population. By meticulously excluding confounding factors such as renal or liver diseases, acute inflammation, and malnutrition, we provide robust evidence of an independent association between hypoalbuminemia, defined as serum albumin levels ≤35 g/L and increased mortality rates, particularly among individuals aged 65 years or older, for both vascular and cancer mortality.

**Implications of all the available evidence**

The implications of our findings extend beyond clinical practice to inform policy and public health strategies, especially for individuals aged 65 years or older. The identification of hypoalbuminemia as a predictor of mortality underscores the importance of routine assessment of serum albumin levels in healthcare settings, particularly within this age group. Additionally, our study highlights the need for further research to explore the potential role of total caloric intake in the observed association between albumin levels and mortality among older adults.

## Introduction

Albumin is synthetized by liver cells and is an important circulating protein, that exerts multiple functions the most important one being the regulation of colloid osmotic pressure.^1^ Albumin, however, has other properties such as transporting proteins, fatty acids or drugs or counteracting inflammation in virtue of its antioxidant property^1^; albumin is in fact rich of thiol groups that allow to quench reactive oxidant species so modulating the redox status.^1^^,^^2^ Albumin encompasses also anticoagulant property by a heparin-like activity and inhibition of clotting factor biosynthesis by liver cells such as factor VIII and V and platelet aggregation inhibition.^1^^,^^3^^,^^4^ In the late eighteen and early nineteen of last century several studies in general population showed that serum albumin inversely correlated with mortality; however, most studies did not exclude confounder factors that may affect serum albumin so limiting conclusive results.^5^, ^6^, ^7^, ^8^, ^9^ More recently a prospective study by the Atherosclerotic Risk in Communities (ARIC) study reported an inverse association between the lower quartile of serum albumin (<39 g/L) and mortality in a population of 4947 individuals followed-up for approximately 5 years.^10^ The peculiarity of the study was in the analysis of some confounding factors that may affect serum albumin including renal disease and inflammation; liver disease and malnutrition, that may also negatively influence the levels of serum albumin, were not analysed.^11^^,^^12^ Further studies tried to assess if serum albumin may intercept cause-specific mortality with data, that, however, are inconclusive. In the case of cancer mortality Philipps et al.^5^ showed that in 7735 men aged 40–59, serum albumin <40 g/L was associated with cancer mortality during a follow-up of 9 years, but confounding factors were not considered; equivocal data on serum albumin and cancer mortality were reported thereafter.^8^^,^^9^^,^^13^ The relationship between serum albumin and cardiovascular disease has been more extensively investigated with data that consistently showed an inverse association between serum albumin and cardiovascular disease (CVD).^13^, ^14^, ^15^, ^16^, ^17^, ^18^ In a large population of 100,899 individuals followed-up for 8.5 years, Ronit et al.^19^ showed that serum albumin values were inversely associated with CVD including myocardial infarction and stroke. In the same report a meta-analysis of 8 studies also confirmed an association between serum albumin and cardiovascular mortality; however, the above reported confounders that may affect serum albumin were incompletely considered. There are, therefore, several caveats in the relationship between serum albumin and mortality, that should be deeper analysed before considering robust and independent such association. An important issue if serum albumin is inversely associated with mortality and cause-specific mortality after excluding factors that may interfere with its blood concentration such as renal and liver disease, acute inflammation and malnutrition; also, it would be relevant for clinical purpose the definition of a specific serum albumin cut-off useable worldwide as measure to identify individuals at higher risk of death. Moreover, considering the serum albumin level at the time of device implantation proves to be of great value when assessing long-term mortality in patients with permanent pacemakers.^20^ Hypoalbuminemia is currently defined as serum albumin ≤35 g/L and occurs prevalently in old population or in patients with frailty or affected by liver or renal disease or chronic inflammation.^1^ In acute clinical settings serum albumin ≤35 g/L is inversely associated with poor survival and CVD in short- and long-term follow-up,^3^^,^^21^^,^^22^ while in general population data regarding total mortality and cause-specific mortality are unclear. Based on this, the aim of the study was to assess if in general population a relationship between hypoalbuminemia, as defined by serum albumin ≤35 g/L, mortality, and cause-specific mortality does exist. To this purpose we prospectively analysed the association of hypoalbuminemia on mortality and cause-specific mortality such as mortality for cancer and vascular disease in a large Italian population included in the Moli-sani study.

## Methods

### Study population

We utilized data from the Moli-sani Study, a population-based cohort established between 2005 and 2010, comprising 24,325 men and women aged ≥35 years residing in the Southern Italian region of Molise. The study's primary objective was to investigate genetic and environmental risk factors contributing to the onset of cardiovascular, cerebrovascular, and tumour diseases. Exclusion criteria encompassed pregnancy during recruitment, mental or decision-making impairments, current polytrauma or coma, and unwillingness to participate. Comprehensive details regarding the Moli-sani Study can be found elsewhere.^23^ Due to their correlation with alterations in albumin levels, individuals with a prior history of cancer or cardiovascular disease or impaired renal function (assessed as estimated Glomerular filtration rate (eGFR) < 30 mL/min/1.73 m^2^) or liver function (assessed with a Fibrosis-4 (Fib-4) score exceeding 3.25 points) or acute inflammation (high sensitivity C-reactive protein (hs-CRP) levels ≥10 mg/L) were excluded from the study. Additionally, participants with missing data on serum albumin, hs-CRP, eGFR, or Fib-4, as well as those with implausible energy intakes (<800 or >4000 kcal/day in men and <500 or >3500 kcal/day in women) or deemed to have unreliable dietary and medical questionnaire responses as assessed by interviewers, were also excluded. A detailed flowchart outlining the selection of study participants is provided in Supplementary Figure S1. The analytical sample consisted of 17,930 individuals.

A randomly selected subset of the entire Moli-sani cohort was recalled for an active follow-up 12 years (median value; range 10–14 years) after the baseline assessment. However, this recall was interrupted due to the COVID-19 pandemic, and only n = 758 individuals, out of the total n = 17,930 participants included in the present study, had serum albumin measurements repeated. It's important to note that this subset of individuals essentially represents a random sample from the entire analysed cohort.

### Exposure and covariates assessment

Diabetes, hypertension, or hyperlipidaemia were ascertained at baseline if participants were taking disease-specific medications. Height and weight measurements were recorded, and body mass index (BMI) was calculated as kg/m^2^. Smoking status was classified as never, current, or former (defined as no smoking within the previous 12 months or more). Leisure-time physical activity was expressed as daily energy expenditure in metabolic equivalent task-hours (MET-h/day) for sport, walking and gardening. Blood pressure was measured using an automatic device (OMRON-HEM-705CP), with three measurements taken on the non-dominant arm, and the average of the last two values was used as the blood pressure reading. Measurements were conducted in a quiet room with a comfortable temperature (from 20 to 23 °C), with participants in a recumbent position for at least 5 min. Educational level was determined by the highest qualification attained and categorized as lower secondary (approximately ≤8 years of education) or upper secondary school (≥9 years). Housing tenure was classified as rented, 1 dwelling ownership and >1 dwelling ownership. Food intake during the year before enrolment was assessed by the EPIC food frequency questionnaire validated and adapted to the Italian population.^24^ Adherence to the traditional Mediterranean diet was assessed through the Mediterranean Diet Score (MDS) developed by Trichopoulou and colleagues,^25^ which was obtained by assigning 1 point to healthy foods (fruits and nuts, vegetables, legumes, fish, cereals, monounsaturated to saturated fats ratio) whose consumption was above the sex-specific medians of intake of the Moli-sani Study population, free from CVD, cancer and diabetes; foods presumed to be detrimental (meat and dairy products) were scored positively if their consumption was below the median. All other intakes received 0 points. Individuals who consumed 10–50 g/day of ethanol for men, and 5–25 g/day for women, from alcoholic beverages of any type were assigned 1 point. Otherwise, the score was 0. The MDS ranged from 0 to 9 (the latter reflecting maximal adherence). The mean intake of proteins was calculated using data from the EPIC questionnaire.

### Laboratory measures

Blood samples were collected at baseline (2005–2010) from participants who had fasted overnight and abstained from smoking for at least 6 h. Lipids (total cholesterol, HDL-cholesterol, triglycerides), and blood glucose were analysed in fresh serum samples using enzymatic reaction methods with an automatic analyser (ILab 350, Instrumentation Laboratory, Milan, Italy). Quality control for lipids and glucose was maintained using two commercial standards, SeraChem® 1 (for normal levels) and SeraChem® 2 (for pathological high levels). The coefficients of variability (CV) for these two commercial standards were 4.9% and 5.2% for blood total cholesterol, 3.2% and 3% for HDL-cholesterol, 5.2% and 5.3% for triglycerides, 4.7% and 4.1% for blood glucose. hs-CRP was measured in fresh serum samples using a particle-enhanced immune-turbidimetric assay (ILab 350, Instrumentation Laboratory, Milan, Italy). Quality control for hs-CRP was maintained using in-house serum pools and commercial laboratory standards, with inter-day coefficients of variability recorded at 4.2% and 5.5% respectively. Subsequently, in frozen samples collected at baseline and follow-up and stored in the Neuromed Biobanking Centre (www.neuromed.it/ricerca/biobanking-centre), serum alanine aminotransferase (ALT), serum aspartate aminotransferase (AST) and creatinine levels were measured using colorimetric enzyme kits, while serum albumin was assessed through a colorimetric assay. All tests in frozen samples were conducted using an automatic analyser (ILab ARIES; Instrumentation Laboratory, Milan, Italy), and quality control was ensured through the use of SeraChem® 1 (for normal levels) and SeraChem® 2 (for pathological high levels) laboratory standards. The CV were 3.9% and 3.6% for ALT, 6.0% and 4.5% for AST, 7.5% and 4.4% for creatinine, 3.4% and 3.6% for serum albumin. Glomerular filtration rate (eGFR) was calculated using the Chronic Kidney Disease Epidemiology Collaboration equation. The FIB-4 score was calculated using the formula: age x AST (IU/l)/platelet count (x109/litre) x square root of (ALT (IU/l)).^26^

### Follow-up for vital status

The Moli-sani Study cohort was followed for mortality from March 2005 to December 31st, 2020, through linkage with the Italian mortality registry ReNCaM (nominative register of causes of death). We assessed cause specific mortality by using the Italian mortality registry, validated by Italian death certificates (ISTAT form) and coded according to ICD-9 (international classification of diseases, revision 9). Vascular mortality included deaths from diseases of the circulatory system when the underlying cause of death included ICD-9 codes 390–459. Cancer death was when the underlying cause of death included ICD-9 codes 140–208. We included non-cardiovascular/noncancer causes of death in an “other cause mortality” group.

### Ethics

The Moli-sani Study adhered to the Declaration of Helsinki and received approval from the Ethics Committee of the Catholic University in Rome, Italy, with ID Prot. pdc. P.99 (A.931/03-138-04)/C.E./2004. The Ethics Committee of the IRCCS Neuromed approved the study's recall. Additionally, written informed consent was obtained from all participants.

### Statistics

The baseline characteristics of participants across serum albumin categories were described as mean ± standard deviation (SD) for continuous variables and frequencies for categorical variables. Differences in the distribution of baseline covariates across serum albumin levels were calculated using generalized linear models adjusted for age and sex. Hazard ratios (HRs) with 95% confidence intervals (95% CI) were used to express the associations between serum albumin levels and all-cause (or cause-specific) mortality. Cox proportional hazards models with time-on-study as the time scale, adjusting for baseline age, were employed for calculations. The proportional hazards assumption was visually assessed using log (−log) plots of survival curves, revealing no violations. Multivariable-adjusted HRs were calculated for three categories of serum albumin levels: ≤35 g/L, 35.1–40.0 g/L, and >40 g/L, with the latter group serving as the reference. Alternatively, serum albumin was treated as a continuous variable, indicating a 1-SD decrease. Potential confounders (measured at baseline) were pre-defined based on existing literature and their documented associations with both serum albumin and mortality, rather than relying solely on statistical criteria.^27^ Two multivariable-adjusted models were employed: a) Model 1 included age (under and below 65 years) and sex; b) Model 2, as in Model 1, further controlled for educational level (two categories), housing (three categories), smoking status (never, current, former smokers), BMI (continuous), adherence to Mediterranean diet (continuous), total calories intake (continuous), percentage of calories from vegetable proteins (continuous), percentage of calories from animal proteins (continuous), leisure time physical activity (continuous), diabetes (no/yes), hypertension (no/yes), hyperlipidaemia (no/yes), hs-CRP (natural log-transformed, continuous), eGFR (continuous), and FIB-4 score (continuous). Participants contributed person-time until the date of death, date of emigration or loss to follow-up, or the end of the follow-up period, whichever occurred first. Participants who died from causes other than those under study were censored at the date of the competing death event. Restricted cubic spline modelling (with 3 knots at 5%, 50%, and 95% of the serum albumin distribution) was employed to explore non-linear associations between serum albumin and mortality.

The primary analysis (Model 2) was replicated within pre-defined subgroups based on sex, age (<65 years and ≥65 years), hypertension, hypercholesterolemia and diabetes. Sensitivity analysis was performed, excluding individuals who experienced events within two years after recruitment. Terms for testing additive interaction, chosen for their effectiveness, were included in the multivariable models to assess variations in the association between serum albumin (three ordered categories) and mortality across population subgroups. This approach provides a robust method for examining whether the combined effects of serum albumin and other variables on mortality differ from what would be expected based on their individual effects alone.^28^ Missing data on covariates (see Supplementary Figure S1 for details) were handled using multiple imputation (SAS PROC MI, followed by PROC MIANALYZE; n = 10 imputed datasets) to maximize data availability. The data analysis was performed using SAS/STAT software, version 9.4 (SAS Institute Inc., Cary, NC, USA), as well as R software, version 4.3.2, from the R Foundation for Statistical Computing, Vienna, Austria. The InteractionR package^29^ in R was utilized for testing additive interaction.

### Role of funding source

This paper was developed within the project funded by Next Generation EU–“Age-It–Ageing well in an ageing society” project (PE0000015), National Recovery and Resilience Plan (NRRP)–PE8–Mission 4, C2, Intervention 1.3”. The views and opinions expressed are only those of the authors and do not necessarily reflect those of the European Union or the European Commission. Neither the European Union nor the European Commission can be held responsible for them. Funders had no role in study design, collection, analysis, and interpretation of data, nor in the writing of the manuscript or in the decision to submit the article for publication. All Authors were and are independent from funders.

## Results

The analytical sample comprised 17,930 individuals, N = 8445 (47.1%) men, with a mean age at enrolment of 54 years (SD = 11 years) and mean serum albumin of 42 g/L (SD = 3.2 g/L). For n = 758 randomly selected individuals, serum albumin analysis was repeated 12.5 years later (ranging from 10.1 to 14.3 years). In this subset of individuals, the distribution of serum albumin at the first and second evaluations was very similar, with means (SD) of 41.8 g/L (2.9 g/L) at baseline and 41.6 g/L (2.7 g/L) at follow-up, respectively (Supplementary Figure S2). The paired P-value for the difference was 0.088.

The entire population was stratified into three serum albumin categories based on predefined criteria and the observed distribution of serum albumin within the population: the “normal” group included the upper two-thirds of the distribution (serum albumin >40 g/L), the “low” group comprised the lowest 2.3% (406/17,930) of the distribution (serum albumin ≤35 g/L), and a “middle” group with serum albumin levels ranging from 35.1 to 40.0 g/L.

Compared to individuals with a normal serum albumin level (>40 g/L), those in the ≤35 g/L category were more likely to be women, older, and had lower social status. They also exhibited higher BMI and hs-CRP levels, lower levels of eGFR and a lower prevalence of hyperlipidaemia (Table 1). Beyond P-values, individuals in different category of serum albumin level displayed only minor variations in adherence to the Mediterranean diet, along with less negligible (if any) differences in calorie intake from proteins (Table 1).

**Table 1:** Main characteristics of the study population (n = 17,930) across categories of serum albumin in the Moli-sani study, Italy (2005–2010).

	All	Serum albumin (g/L)			P-value^a^
		≤35.0	35.1–40.0	>40.0	
N of individuals (n, %)	17,930 (100.0)	406 (2.3)	5466 (30.5)	12,058 (67.2)	–
Serum albumin (g/L)	42 ± 3	34 ± 2	39 ± 1	44 ± 2	–
Men (%)	47.1	33.7	37.2	52.0	<0.0001
Age (years)	54 ± 11	56 ± 12	56 ± 12	53 ± 11	<0.0001
Age ≥65 years (%)	18.4	29.1	22.9	16.0	<0.0001
Educational level (%)					0.041
Up to lower secondary	50.8	52.7	54.3	49.2	
Upper secondary	49.2	47.3	45.7	50.8	
Housing categories (%)					0.015
Rented	8.6	10.3	8.3	8.7	
1 dwelling ownership	82.9	78.8	83.8	82.6	
>1 dwelling ownership	8.5	10.8	7.8	8.7	
Smoking status (%)					0.0020
Non-smokers	50.3	54.7	52.5	49.2	
Current	23.7	23.4	23.9	23.6	
Former	26.0	21.9	23.6	27.2	
Leisure-time physical activity (MET-h/day)	3.5 ± 4.0	3.2 ± 3.6	3.3 ± 4.0	3.7 ± 4.1	0.072
BMI (kg/m^2^)	27.8 ± 4.6	28.5 ± 5.2	28.2 ± 4.9	27.7 ± 4.4	<0.0001
Mediterranean Diet Score (points)	4.4 ± 1.6	4.5 ± 1.6	4.3 ± 1.6	4.4 ± 1.6	<0.0001
% of calories from vegetable proteins (%)	5.5 ± 0.9	5.5 ± 0.9	5.4 ± 0.9	5.5 ± 0.9	0.032
% of calories from animal proteins (%)	10.8 ± 2.5	10.8 ± 2.5	10.9 ± 2.5	10.7 ± 2.5	0.16
% of calories from total proteins (%)	16.2 ± 2.1	16.3 ± 2.2	16.4 ± 2.2	16.1 ± 2.1	0.54
Total calories intake (Kcal)	2098 ± 573	1996 ± 570	2047 ± 569	2124 ± 572	0.44
Diabetes (%)	8.0	8.1	8.1	8.0	0.73
Hypertension (%)	53.9	54.9	53.2	54.2	<0.0001
Hyperlipidaemia (%)	29.9	20.2	27.8	31.2	<0.0001
hs-CRP (mg/L)	2.0 ± 1.9	2.5 ± 2.3	2.2 ± 2.0	1.9 ± 1.8	<0.0001
Fib-4 score (points)	1.2 ± 0.5	1.3 ± 0.6	1.3 ± 0.5	1.2 ± 0.5	0.95
eGFR (mL/min/1.73 m^2^)	93 ± 14	92 ± 15	93 ± 14	93 ± 14	<0.0001

a Multivariable comparison across ordered serum albumin categories, adjusted for age and sex. Continuous or categorical variables are expressed as mean values and standard deviation or percentage, respectively. Abbreviation: BMI, body mass index; hs-CRP, high-sensitivity C-reactive protein; Fib-4, Fibrosis-4 score; eGFR, estimated Glomerular Filtration Rate.

### Association with mortality

Among the 1428 all-cause deaths that occurred over a median follow-up of 13.1 years (interquartile range: 12.2–14.2 years; 231,394 person-years), 464 deaths were attributed to vascular disease, 574 to cancer, and 390 to other causes.

**Fig. 1:**
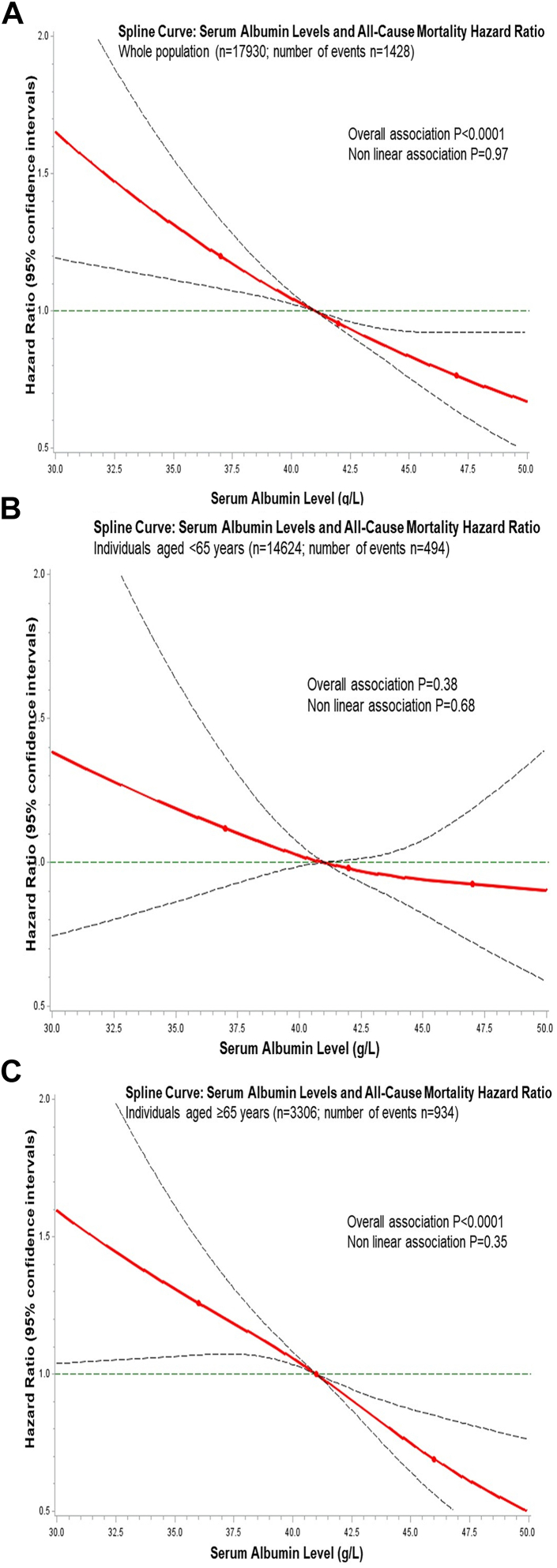
Spline curves for the association of serum albumin levels with all-cause mortality. Panel A) whole cohort; panel B) individuals aged 35–65 years; panel C) individuals aged ≥65 years. Multivariable dose–response association of all-cause mortality with serum albumin levels (g/L) amongst participants from the Moli-sani Study cohort in Italy (2005–2010), in the whole cohort and according to age. Hazard ratios with 95% confidence interval were obtained from a multivariable model adjusted for age (when not stratified for), sex, educational level, housing, smoking status, BMI, adherence to Mediterranean diet, total calories intake, percentage of calories from vegetable proteins, percentage of calories from animal proteins, leisure time physical activity, history of diabetes, hypertension, hyperlipidaemia, log (hs-CRP), FIB-4 score and eGFR. The dashed lines indicate 95% confidence bands. 3 knots were used, located at the 5th, 50th, and 95th percentiles of the serum albumin distribution. Reference value was fixed at 41 g/L (median value of the serum albumin distribution). Analyses were generated using the first imputed dataset. The other imputed datasets were similar and thus omitted.

The multivariable (adjusted for sociodemographic, nutritional, and clinical factors) dose–response analysis of serum albumin with all-cause mortality revealed an inverse linear dose–response relationship (P-value for the overall association <0.0001, and P-value for non-linearity = 0.97) (Fig. 1A), indicating that individuals with serum albumin levels below the reference value of 41 g/L displayed a linear increasing rate of mortality as serum albumin levels decreased.

Compared to individuals with normal serum albumin values, the multivariable hazard ratios (HRs) for all-cause mortality for those with serum albumin levels between 35.1 and 40.0 g/L and ≤35 g/L were 1.23 (95% CI: 1.10–1.37) and 1.61 (95% CI: 1.21–2.13), respectively (Table 2). When serum albumin was analysed as a continuous variable, a 1-SD decrease (SD = 3.2 g/L) was associated with a 16% higher mortality rate (95% CI: 9%–22%).

**Table 2:** All-cause and cause specific mortality hazard ratios across baseline categories of serum albumin in the Moli-sani study (2005–2010).

	Serum albumin (g/L)			
	≤35	35.1–40	>40	1-SD decrease^a^
**All-cause mortality** (total number of events n = 1428)				
N of deaths/n of individuals	52/406	536/5466	840/12,058	–
Person-years, n	5135	69,566	156,693	–
Event rates per 10,000 person-years	101	77	54	–
Model 1 HR (95% CI)	1.55 (1.17–2.06)	1.28 (1.14–1.42)	-1-	1.17 (1.11–1.23)
Model 2 HR (95% CI)	1.61 (1.21–2.13)	1.23 (1.10–1.37)	-1-	1.16 (1.09–1.22)
**Vascular mortality** (total number of events n = 464)				
Model 2 HR (95% CI)	1.40 (0.84–2.33)	1.25 (1.03–1.51)	-1-	1.16 (1.05–1.28)
**Cancer mortality** (total number of events n = 574)				
Model 2 HR (95% CI)	1.38 (0.85–2.26)	1.25 (1.05–1.49)	-1-	1.13 (1.03–1.23)
**Mortality for other causes** (total number of events n = 390)				
Model 2 HR (95% CI)	2.23 (1.39–3.58)	1.16 (0.94–1.44)	-1-	1.20 (1.08–1.33)

a Serum albumin SD = 3.2 g/L. Hazard ratios (HR) with 95% confidence interval (95% CI) obtained from multivariable cause-specific Cox proportional hazards regression models. Model 1 was adjusted for sex and age (under and over 65 years). Model 2 was adjusted for sex, age (under and over 65 years), educational level, housing, smoking status, body mass index, adherence to Mediterranean diet, total calories intake, percentage of calories from vegetable proteins, percentage of calories from animal proteins, leisure time physical activity, history of diabetes, hypertension, hyperlipidaemia, natural log (C-reactive protein), Fibrosis-4 score and estimated Glomerular Filtration Rate.

The same trend of a higher rate of deaths at lower serum albumin levels was observed for vascular, cancer, or other causes of mortality (Table 2). The trend was more pronounced for causes of mortality that were neither vascular nor cancer-related (Table 2).

After excluding deaths that occurred within 2 years of recruitment (n = 75), the association between low serum albumin levels and overall mortality showed a consistent pattern (HR per 1-SD decrease: 1.15; 96% CI: 1.09–1.22), which was also observed for specific causes of death.

### Subgroup analyses

The association of serum albumin with mortality was equally observed in men and women (P for difference = 0.071, P = 0.41, P = 0.015, and P = 0.44 for total, vascular, cancer, and other causes of mortality, respectively). Similarly, stratification for hypertension, hypercholesterolemia, or diabetes showed no differences in effect (P > 0.15, considering any comorbidity or type of event).

On the contrary, a clear difference in the association of serum albumin with mortality according to age was observed. Specifically, the association was null when restricted to individuals aged <65 (Fig. 1B and Table 3) but evident in individuals aged 65 years or older (Fig. 1C and Table 3). Compared to individuals with normal serum albumin values (>40 g/L), the multivariable hazard ratios (HRs) for all-cause mortality for those with serum albumin levels ≤35 g/L were 1.83 (95% CI: 1.33–2.50) in individuals aged ≥65 years and 1.03 (95% CI: 0.53–2.00) in those aged <65 years (Table 3; P for difference <0.0001 when additive interaction was calculated considering serum albumin in ordered categories). A 1-SD lower value in serum albumin was associated with a 20% (HR = 1.20; 95% CI: 1.12–1.29) and 6% (HR = 1.06; 95% CI: 0.97–1.17) higher mortality rate in older and younger individuals, respectively (Table 3).

**Table 3:** All-cause and cause specific mortality hazard ratios across baseline categories of serum albumin and according to age in the Moli-sani study (2005–2010).

	Sample size/No. of event	Serum albumin (g/L)			P for difference	1-SD decrease^a^
		≤35	35.1–40	>40		
		HR (95% CI)	HR (95% CI)	Referent		HR (95% CI)
**All-cause mortality**						
Age 35–65 years	494/14,624	1.03 (0.53–2.00)	1.12 (0.92–1.37)	-1-	<0.0001	1.06 (0.97–1.17)
Age ≥65 years	934/3306	1.83 (1.33–2.50)	1.26 (1.10–1.44)	-1-		1.20 (1.12–1.29)
**Vascular mortality**						
Age 35–65 years	100/14,624	1.57 (0.49–5.03)	0.99 (0.63–1.56)	-1-	0.0082	1.05 (0.86–1.29)
Age ≥65 years	364/3306	1.38 (0.78–2.43)	1.31 (1.06–1.62)	-1-		1.19 (1.07–1.33)
**Cancer mortality**						
Age 35–65 years	280/14,624	1.04 (0.43–2.53)	1.16 (0.90–1.51)	-1-	0.046	1.08 (0.96–1.23)
Age ≥65 years	294/3306	1.55 (0.86–2.81)	1.30 (1.02–1.65)	-1-		1.15 (1.02–1.30)
**Mortality for other causes**						
Age 35–65 years	114/14,624	0.50 (0.07–3.58)	1.15 (0.76–1.72)	-1-	0.0070	1.02 (0.84–1.16)
Age ≥65 years	276/3306	2.83 (1.72–4.67)	1.15 (0.89–1.48)	-1-		1.27 (1.12–1.44)

a Serum albumin SD = 3.2 g/L. Hazard ratios (HR) with 95% confidence interval (95% CI) obtained from multivariable cause-specific Cox proportional hazards regression models. Model adjusted for sex, educational level, housing, smoking status, body mass index, adherence to Mediterranean diet, total calories intake, percentage of calories from vegetable proteins, percentage of calories from animal proteins, leisure time physical activity, history of diabetes, hypertension, hyperlipidaemia, log (C-reactive protein), Fibrosis-4 score and estimated Glomerular Filtration Rate.

A similar trend was observed for the three different causes of death considered, with a higher rate of mortality associated with lower serum albumin levels that was nearly absent in younger individuals but manifest in older individuals (Table 3).

As expected, individuals aged less than or more than 65 years exhibited differences in all the characteristics considered (Supplementary Table S1). It is noteworthy that the prevalence of hypoalbuminemia (serum albumin ≤35 g/L) was higher among individuals aged 65 years or older (118/3306, 3.6%) compared to those under 65 years of age (288/14,624, 1.0%; P < 0.0001). Nevertheless, beyond P-values, it is worth noting that the two groups displayed only minor difference in the adherence to the Mediterranean diet (0.2 point higher when age ≥65 years), along with less negligible differences in calorie intake from proteins (Supplementary Table S1). In contrast, total calorie intake was considerably lower in individuals aged 65 years or older compared to younger population (1864 Kcal versus. 2151 Kcal, respectively).

## Discussion

This study offers evidence that in an ostensibly healthy general adult population, individuals with hypoalbuminemia (defined as serum albumin levels ≤35 g/L) experienced a higher mortality rate. This was observed independently of factors that might influence serum albumin levels, such as liver or renal disease, acute inflammation, or nutritional status. Specifically, hypoalbuminemia identified individuals at an elevated risk of mortality from various causes, including cancer and vascular-related deaths. Notably, this increased risk was evident only for those aged ≥65 years.

In our population living in the South of Italy, 2.3% of individuals displayed hypoalbuminemia; this laboratory feature was observed more frequently in individuals aged 65 years or older, as well as in those exhibiting low-grade systemic inflammation. To the best of our knowledge, this is the largest study in a general population exploring the relation between serum albumin and mortality and between serum albumin and cause-specific mortality such as mortality for cancer and vascular disease. Even if our report is consistent with previous studies reporting an inverse association between serum albumin and total mortality, the peculiarity of our finding is in the choice of a specific serum albumin cut-off potentially useable worldwide for identifying people at risk and in the analysis of serum albumin versus mortality after exclusion of known factors influencing serum albumin. Thus, our findings were derived from a population not including individuals with a personal history of renal or liver disease, that may reduce serum albumin by enhancing its excretion or lowering its biosynthesis, respectively. Furthermore, individuals with history of cardiovascular disease or cancer or acute systemic inflammation -conditions typically associated with decreased serum albumin levels-were also excluded from the study.^1^ More relevant is the fact that our analysis revealed no substantial differences in the nutritional profile (as assessed by adherence to the Mediterranean diet and the percentage distribution of total calories derived from total proteins, categorized into animal and plant sources) among various categories of individuals with decreasing levels of serum albumin. However, despite these similarities, a significant distinction in total calorie intake emerged, with individuals characterized by lower albumin levels exhibiting an overall reduced caloric intake. This suggests the possibility that the disparity in total calorie intake might contribute, at least in part, to the observed association between low albumin levels and an increased mortality rate, although the role of total caloric intake in mortality is unclear. Additional studies are essential to examine and validate the specific role of total caloric intake in the identified association between albumin levels and mortality. Implementing precise controls for calorie intake in future research will be crucial for isolating albumin's independent contribution to mortality outcomes. Investigating the intricate interplay between albumin and mortality, while considering various confounding factors, including total calorie intake, will contribute to a comprehensive understanding of the underlying mechanisms.

Moreover, our findings indicated an independent association between low serum albumin levels and an increased rate of mortality among individuals aged 65 years or older, while such an association was notably absent in younger individuals. Importantly, our analysis of nutritional profiles revealed no differences between the two age groups, underscoring the consistency in dietary patterns. The only notable distinction lies in total calories intake, which is higher among the younger cohort. This observation prompts the consideration that the age-related difference in the impact of low albumin levels on mortality cannot be attributed to a divergent nutritional profile, given the lack of substantial variations, except for differences in total caloric intake. Conversely, we observed a higher prevalence of hypoalbuminemia in individuals aged 65 years or older reinforcing previous reports showing an inverse association between serum albumin and elderly population.^6^^,^^7^^,^^10^

While our data support in part previous findings reporting an association between serum albumin and cardiovascular mortality^5^, ^6^, ^7^, ^8^, ^9^, ^10^, ^11^, ^12^ indicating, in particular, that such relationship is independent from confounding factors and detectable only in old population, the relationship between serum albumin and cancer-related mortality in old population is novel, if compared to the scarce and equivocal data so far reported. After the first study by Phillipps et al. showing that men with serum albumin<40 g/L were at higher risk of cancer mortality,^5^ two following studies including 1754^8^ and 939^13^ men did not confirm such association. Conversely, in 6957 individuals aged over 65 years followed-up for 13.7 years, Okamura et al.^9^ reported that serum albumin was significantly associated with cancer mortality in a population younger than 65 years; however, adjustment for confounding factors was not reported, thereby it is difficult to explain the disagreement of this study with our report. In the context of hypoalbuminemia and cancer, it is also of interest that in a large population (n = 100,122) serum albumin<35 g/L was associated with incident cancer during a follow-up of 1 year; such association was adjusted for liver disease and nephrotic syndrome but no data on cancer mortality were provided.^30^

The fact that hypoalbuminemia is inversely associated with both cancer and vascular mortality provides support to the hypothesis that cancer and cardiovascular disease may share similar disease mechanism.^31^, ^32^, ^33^, ^34^, ^35^ There is, in fact, a growing body of evidence that risk factors such as obesity, diabetes mellitus and dyslipidaemia, that are characterized by systemic inflammation, may be complicated by cardiovascular disease as well as cancer.^33^, ^34^, ^35^, ^36^, ^37^, ^38^, ^39^ Of note, oxidative stress consequent to overproduction of reactive oxidant species is a typical feature of cancer and cardiovascular disease^33^ and the fact that a decrease of a powerful antioxidant protein such as serum albumin is associated with cancer and cardiovascular mortality provides further support to the potentially pathogenic role of redox status in both diseases.^33^^,^^34^ In this context, aging may represent an important factor predisposing to oxidative stress and eventually cancer and vascular disease, which, in fact, are more frequent in elderly population.^32^^,^^40^ Thus, aging is associated with overactivation of enzymes producing reactive oxidant species such as Nox2 or Myeloperoxidase along with a decline of antioxidants such as Catalase or Glutathione Peroxidase 3 resulting in an oxidative stress consequent to an imbalance between oxidant molecule production and antioxidant defence.^41^ The association between aging and hypoalbuminemia, a potent antioxidant, underscores the notion that oxidative stress frequently accompanies aging. This oxidative stress may reflect the low-grade inflammation characteristic of cardiovascular and cancer diseases.^31^, ^32^, ^33^ Consistent with this perspective, our findings indicate an inverse relationship between serum albumin and hs-CRP. This suggests that early detection of hypoalbuminemia could serve as an indicator of low-grade systemic inflammation, signalling potential cardiovascular and cancer risks.

This study has implications and limitations. We used a specific serum albumin cut-off to identify individuals at risk of mortality and cause-specific mortality, that is a very cheap laboratory analysis and easily useable worldwide. The clinical relevance of our reports relies on the fact that such association is independent on confounders and that analysis of serum albumin is a reliable assay as documented by its low variability in samples taken in two separate periods. Serum albumin levels are scarcely considered in clinical practise unless patients have circulatory disturbances related to oncotic pressure.^1^ In other clinical conditions hypoalbuminemia is often overlooked as it is regarded as a phenomenon secondary the primary pathology causing its reduction.^42^ Conversely, our data indicate that serum albumin levels should be considered not only as measure of oncotic pressure or epiphenomenon of underlying disease but also as a variable indicating a risk for vascular disease and cancer. Further studies could explore whether combining albumin with other laboratory variables or utilizing a nutritional score might enhance its predictive value.^43^^,^^44^

Aging seems to greatly contribute to hypoalbuminemia and the coexistent low-grade inflammation may represent a key factor. The concept of *inflammaging* has been recently coined to outline the importance of inflammation as trigger of disease in elderly^32^; further study is necessary to establish if hypoalbuminemia reflects multiple or specific cause-related inflammation. Hypoalbuminemia was more frequent in women, that is consistent with a report from the ARIC study on the same topic^10^; further study is, however, needed for elucidating this sex-related serum albumin behaviour in the general population. We have not established data to explain why individuals with low income exhibit lower serum albumin levels. However, it cannot be ruled out that the quality of food might be poorer among low-income individuals, given that healthy foods are typically more expensive.^45^ Nevertheless, our results remain unaffected, as we adjusted for socioeconomic status indicators.

A challenging aspect to address was the connection detected between hypoalbuminemia and mortality for causes other than vascular or cancer-related issues. This finding included a wide range of deaths associated with conditions like renal, pulmonary, or gastrointestinal diseases; due to the limited number of events for each specific cause of death, it is difficult to determine if hypoalbuminemia is also a factor associated with other causes of mortality. Further study should be done to explore the interplay between hypoalbuminemia and non-vascular/non-cancer deaths.

While our study benefits from a prospective design and a substantial sample size, inherent limitations associated with observational studies must be acknowledged. Establishing causal relationships remains challenging, and despite efforts to control for confounding variables, unmeasured or residual confounding may still impact the observed associations.^46^ A cause–effect relationship should be investigated by appropriate prospective study to establish if increasing serum albumin may improve clinical outcomes. Additionally, the absence of randomization introduces the potential for selection bias, and the reliance on self-reported or recorded data may introduce measurement error. Furthermore, the generalizability of our findings may be influenced by the characteristics of the study population. Thus, the study has been conducted in a Caucasian population living in the South of Italy, further study should be done to see if this finding is replicated in other countries and races. These inherent limitations underscore the need for cautious interpretation and highlight the importance of further research, potentially employing experimental designs, to validate and extend our observations. In addition, it is important to note that both the exposure (serum albumin levels) and covariates were measured only once at baseline, which represents another limitation of our study, as it may not capture potential changes in these variables over time. However, it is noteworthy that within the sub-sample where albumin measurements were available at two time points, values remained remarkably consistent, providing some reassurance regarding the stability of the measured levels.

Another potential limitation involves the use of self-reported data for lifestyle factors, which may introduce misclassification bias. It is essential to note that we diligently addressed potential biases in reporting clinical conditions at baseline. Individuals reporting specific medical events, such as cardiovascular disease, were required to provide verifiable details about the treating clinical facility, and those reporting medication usage were requested to present the corresponding packaging for confirmation. However, despite these efforts, the inherent limitations of relying solely on self-reported data persist. While we recognize this as a constraint, we believe our rigorous verification procedures for clinically relevant information help mitigate the potential impact of misclassification bias. This approach aligns with the challenges faced by many large-scale population studies.

While our cohort includes individuals of diverse ages, ranging from 35 years and above, the observed association between low albumin levels and increased mortality is particularly evident in those aged 65 and above. We acknowledge the potential influence of survival bias, especially among the older individuals in the cohort. Despite efforts to address biases through statistical methods, the inherent challenges associated with survival bias in observational studies are recognized.

In conclusion, our study reveals that hypoalbuminemia is associated with overall mortality and cause-specific mortality, including deaths from cancer and vascular disease, in individuals aged 65 years or older. Analysis of serum albumin may represent a simple and cheap tool to identify individuals with poor survival.

## Contributors

FV and ADiC conceived and designed the study. MB, SC, MP, TP and FB acquired the data. SC and ADiC managed the data. ADeC and SM measured biomarkers. ADiC analysed the data. ADiC and FV drafted the manuscript. PP, RC, CC, MBD, GdG and LI critically revised this manuscript. CC, MBD, GdG and LI originally promoted the Moli-sani study. LI, ADiC and SC have accessed and verified the data. LI, FV, ADiC and GdG were responsible for the decision to submit the manuscript. The final two authors (LI and FV) are listed as co-last authors. The Moli-sani Study Investigators are presented in the supplementary file. All Authors read and approved the final version of the manuscript and agreed to be accountable for all aspects of the work ensuring integrity and accuracy.

## Data sharing statement

The data underlying this article will be shared on reasonable request to the corresponding author. The data are stored in an institutional repository (https://repository.neuromed.it) and access is restricted by the ethical approvals and the legislation of the European Union.

## Declaration of interests

The Authors declare no conflict of interest.
