# Supplementary material for: The association between hypoalbuminemia and risk of death due to cancer and vascular disease in individuals aged 65 years and older: findings from the prospective Moli-sani cohort study
Source: eClinicalMedicine. 2024 May 8;72:102627. doi: 10.1016/j.eclinm.2024.102627 (PMC11247150; doi:10.1016/j.eclinm.2024.102627)
Supplement: Supplemental file 2 [file mmc1.docx]

**Supplementary material**

**Supplementary Table 1** Main characteristics of the study population (n=17,930) across age categories

|  | **Age** | |
| --- | --- | --- |
|  | **35 to 65 years** | **≥65 years** |
| N of individuals (n, %) | 14,624 (81.6) | 3,306 (18.4) |
| Serum albumin, mean value (g/L) | 42.0±3.2 | 41.0±3.0 |
| Serum albumin >40 g/L (n, %) | 10,123 (69.2) | 1,935 (58.5) |
| Serum albumin 35.1 to 40 g/L (n, %) | 4,213 (28.8) | 1,253 (37.9) |
| Serum albumin ≤35 g/L (n, %) | 288 (1.0) | 118 (3.6) |
| Men (%) | 47.0 | 47.6 |
| Age (±SD; years) | 50±8 | 72±5 |
| Educational level (%) |  |  |
| Up to lower secondary | 45.4 | 74.8 |
| Upper secondary | 54.6 | 25.2 |
| Housing categories (%) |  |  |
| Rented | 9.2 | 6.1 |
| 1 dwelling ownership | 82.6 | 84.1 |
| >1 dwelling ownership | 8.2 | 9.8 |
| Smoking status (%) |  |  |
| Non-smokers | 48.5 | 58.3 |
| Current | 26.7 | 10.6 |
| Former | 24.8 | 31.1 |
| Leisure-time physical activity (±SD; MET-h/day) | 3.5±3.9 | 3.9±4.6 |
| BMI (±SD; kg/m^2^) | 27.6±4.6 | 28.7±4.6 |
| Mediterranean Diet Score (±SD; points) | 4.3±1.6 | 4.5±1.6 |
| Percentage of calories from vegetable proteins (±SD; %) | 5.4±0.9 | 5.6±1.0 |
| Percentage of calories from animal proteins (±SD; %) | 10.8±2.4 | 10.7±2.6 |
| Percentage of calories from total proteins (±SD; %) | 16.2±2.1 | 16.3±2.3 |
| Total calories intake (±SD; Kcal/d) | 2,151±567 | 1,864±539 |
| Diabetes (%) | 6.4 | 15.3 |
| Hypertension (%) | 46.4 | 86.8 |
| Hyperlipidaemia (%) | 28.5 | 35.9 |
| hs-CRP (±SD; mg/L) | 1.9±1.8 | 2.5±2.0 |
| Fib-4 score (±SD; points) | 1.1±0.4 | 1.8±0.5 |
| eGFR (±SD; mL/min/1.73m^2^) | 96±12 | 80±12 |

Sex-adjusted P-values for differences were <0.0001 for all the characteristics reported in the Table, except for the percentage of calories from animal proteins (P=0.071) and the percentage of calories from total proteins (P=0.033). Abbreviation: BMI, body mass index: hs-CRP, high-sensitivity C-reactive protein; Fib-4, Fibrosis-4 score; eGFR, estimated Glomerular Filtration Rate.

**Supplementary Figure legends**

**Supplementary Figure 1** Flowchart of the study. The groups of removed participants (out of the 24,325 recruited at baseline) are overlaid. The final study sample cannot be calculated as a subtraction of the sum of eliminated groups out of the recruited individuals at baseline.

**Supplementary Figure 2** The distribution of serum albumin was measured at baseline and again at follow-up in the same individuals (N=758). As depicted, a large portion of the measurements overlap, as indicated by the data shown in middle-grey.

**Supplementary Figure 1**

**
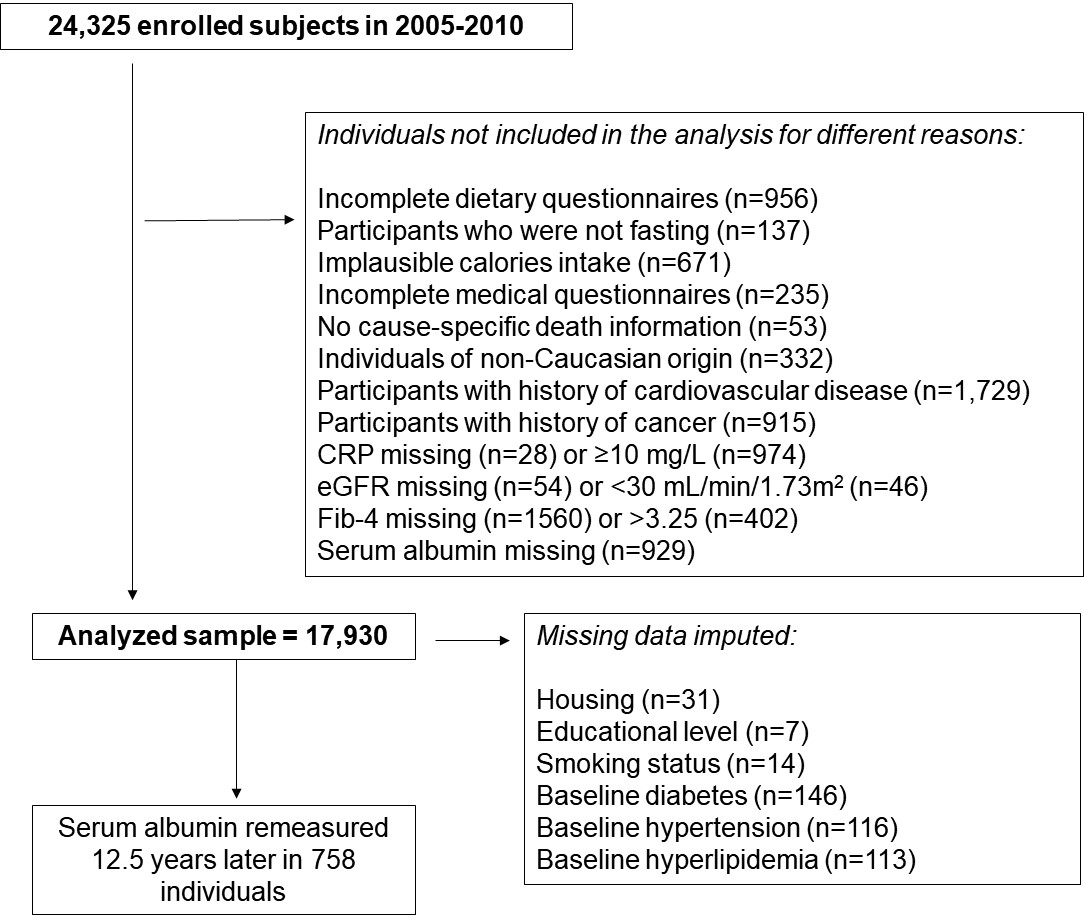
**

**Supplementary Figure 2**


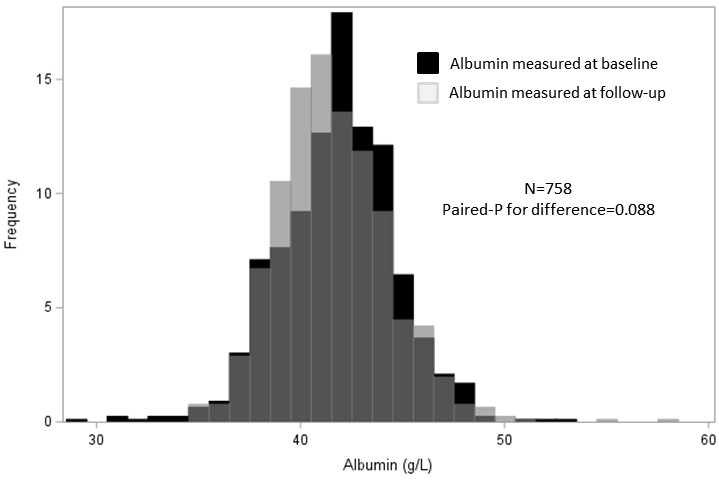


**Moli-sani Study Investigators**

The enrolment phase of the Moli-sani Study was conducted at the Research Laboratories of the Catholic University in Campobasso (Italy), the follow up of the Moli-sani cohort is being conducted at the Department of Epidemiology and Prevention of the IRCCS Neuromed, Pozzilli, Italy.

**Steering Committee:** Licia Iacoviello*^#^ (chairperson), Giovanni de Gaetano* and Maria Benedetta Donati*.

**Scientific Secretariat:** Chiara Cerletti* (coordinator), Marialaura Bonaccio*, Americo Bonanni*, Simona Costanzo*, Amalia De Curtis*, Augusto Di Castelnuovo^§^, Alessandro Gialluisi*^#^, Francesco Gianfagna°^§^, Mariarosaria Persichillo*, Teresa Di Prospero* (secretary).

**Safety and Ethical Committee:** Jos Vermylen (Catholic University, Leuven, Belgium) (Chairperson), Renzo Pegoraro (Pontificia Accademia per la Vita, Roma, Italy), Antonio Spagnolo (Catholic University, Roma, Italy).

**External Event Adjudicating Committee**: Deodato Assanelli (Brescia, Italy), Livia Rago (Campobasso, Italy).

**Baseline and Follow-up Data Management:** Simona Costanzo* (coordinator), Marco Olivieri (Campobasso, Italy), Sabatino Orlandi*, Teresa Panzera*.

**Data Analysis:** Augusto Di Castelnuovo^§^ (coordinator), Marialaura Bonaccio*, Simona Costanzo*, Simona Esposito*, Alessandro Gialluisi*^#^, Anwal Ghulam*, Francesco Gianfagna°^§^, Roberta Parisi, Antonietta Pepe*, Emilia Ruggiero*, Francesca Bracone*, Sukshma Sharma*.

**Biobank, Molecular and Genetic Laboratory:** Amalia De Curtis* (Coordinator), Concetta Civitillo*, Alisia Cretella*, Sara Magnacca^§^, Fabrizia Noro*.

**Recruitment Staff:** Mariarosaria Persichillo* (coordinator), Francesca Bracone*, Giuseppe Di Costanzo*, Sabrina Franciosa*, Martina Morelli*, Teresa Panzera*.

**Communication and Press Office:** Americo Bonanni*.

**Regional Institutions:** Direzione Generale per la Salute - Regione Molise; Azienda Sanitaria Regionale del Molise (ASReM, Italy); Agenzia Regionale per la Protezione Ambientale del Molise (ARPA Molise, Italy); Molise Dati Spa (Campobasso, Italy); Offices of vital statistics of the Molise region.

**Hospitals:** Presidi Ospedalieri ASReM: Ospedale A. Cardarelli – Campobasso, Ospedale F. Veneziale – Isernia, Ospedale San Timoteo - Termoli (CB), Ospedale Ss. Rosario - Venafro (IS), Ospedale Vietri – Larino (CB), Ospedale San Francesco Caracciolo - Agnone (IS); Casa di Cura Villa Maria - Campobasso; Responsible Research Hospital - Campobasso; IRCCS Neuromed - Pozzilli (IS).

***Department of Epidemiology and Prevention, IRCCS Neuromed, Pozzilli, Italy

^#^Department of Medicine and Surgery, LUM University “Giuseppe Degennaro”, Casamassima, Italy

^§^Mediterranea Cardiocentro, Napoli, Italy

°Department of Medicine and Surgery, University of Insubria, Varese, Italy

*Moli-sani Study Past Investigators are available at* [*https://www.moli-sani.org/?page_id=173*](https://www.moli-sani.org/?page_id=173)

| **First names** | **Surnames** |
| --- | --- |
| Deodato | Assanelli |
| Marialaura | Bonaccio |
| Americo | Bonanni |
| Francesca | Bracone |
| Chiara | Cerletti |
| Concetta | Civitillo |
| Simona | Costanzo |
| Alisia | Cretella |
| Amalia | De Curtis |
| Giovanni | de Gaetano |
| Augusto | Di Castelnuovo |
| Giuseppe | Di Costanzo |
| Teresa | Di Prospero |
| Maria Benedetta | Donati |
| Simona | Esposito |
| Sabrina | Franciosa |
| Anwal | Ghulam |
| Alessandro | Gialluisi |
| Francesco | Gianfagna |
| Licia | Iacoviello |
| Sara | Magnacca |
| Martina | Morelli |
| Fabrizia | Noro |
| Marco | Olivieri |
| Sabatino | Orlandi |
| Teresa | Panzera |
| Roberta | Parisi |
| Renzo | Pegoraro |
| Antonietta | Pepe |
| Mariarosaria | Persichillo |
| Livia | Rago |
| Emilia | Ruggiero |
| Sukshma | Sharma |
| Antonio | Spagnolo |
| Jos | Vermylen |
